# Bridging the digital divide: student-led literacy initiatives in diabetes management

**DOI:** 10.3389/fcdhc.2025.1734776

**Published:** 2025-12-02

**Authors:** Pedro Almeida Moyano, Mohammed Raddaoui, Andrea de Barros Coscelli Ferraz, Gustavo José Martiniano Porfírio, Luciana Aparecida Campos, Ovidiu Constantin Baltatu

**Affiliations:** 1 Center of Innovation, Technology, and Education (CITE) at Anhembi Morumbi University, Anima Institute, Sao Jose dos Campos, Brazil; 2 Inspirali Research Organization (IRO), Inspirali, Anima Educação, São Paulo, Brazil; 3 College of Medicine, Alfaisal University, Riyadh, Saudi Arabia

**Keywords:** digital health, diabetes management, digital divide, health equity, digital literacy, student-led initiatives, digital natives, intergenerational mentoring

## Abstract

The rapid advancement of digital health technologies—such as continuous glucose monitors, automated insulin delivery systems, and telehealth platforms—has transformed diabetes management. However, a persistent digital divide continues to amplify health disparities based on socioeconomic status, geography, and age. Generational gaps are especially notable. “Digital immigrants” (older adults) often face significant barriers to technology adoption, such as reduced digital literacy, lower smartphone ownership, and difficulties using remote care platforms. Individuals aged 80 and above have much lower uptake of continuous glucose monitors and telemedicine, even when cost is not a barrier. Health literacy and language challenges further increase the impact and reduce the use of digital health tools among older adults. In contrast, “digital natives”—younger generations with strong technological skills—are well positioned to help bridge this divide. This mini-review summarizes current evidence on disparities in digital diabetes care and proposes an innovative, student-led solution. We advocate for empowering digital native health sciences students to lead digital health literacy initiatives and serve as technology mentors for both patients and clinicians. By establishing student-led digital literacy centers, academic institutions can promote intergenerational collaboration. This approach can turn the generational divide from a barrier into an opportunity. Ultimately, student-led initiatives offer a sustainable, community-based pathway to equitable adoption of digital diabetes technologies and improved health outcomes.

## 1 Introduction

Diabetes mellitus is a rapidly escalating global health crisis. In 2021, an estimated 536.6 million adults worldwide were affected. This represents a prevalence of 10.5% among adults aged 20–79 years. Projections show that the number of adults with diabetes will rise to nearly 590 million by 2025 and 783 million by 2045 (1–3). The economic burden is also substantial. Global health expenditures related to diabetes reached $966 billion USD in 2021 and are expected to surpass $1 trillion by 2045 (1, 2).

To address this crisis, digital health technologies have become important tools for diabetes management. Innovations now include continuous glucose monitoring (CGM), mobile health (mHealth) applications, telemedicine platforms, AI-driven decision support for both clinicians (e.g., treatment recommendations, risk prediction) and patients (e.g., personalized lifestyle guidance, dosing calculators), and integrated digital management platforms. These technologies are increasingly used to improve glycemic control and streamline healthcare delivery through data monitoring and sharing, and enhance patient self-monitoring behaviors through real-time feedback and data visualization (4–8). Recent research shows that these technologies can significantly reduce HbA1c levels. They also help improve self-management behaviors and provide cost-effective solutions for both type 1 and type 2 diabetes (9, 10).

## 2 Digital health technologies in diabetes management

**Figure 1:**
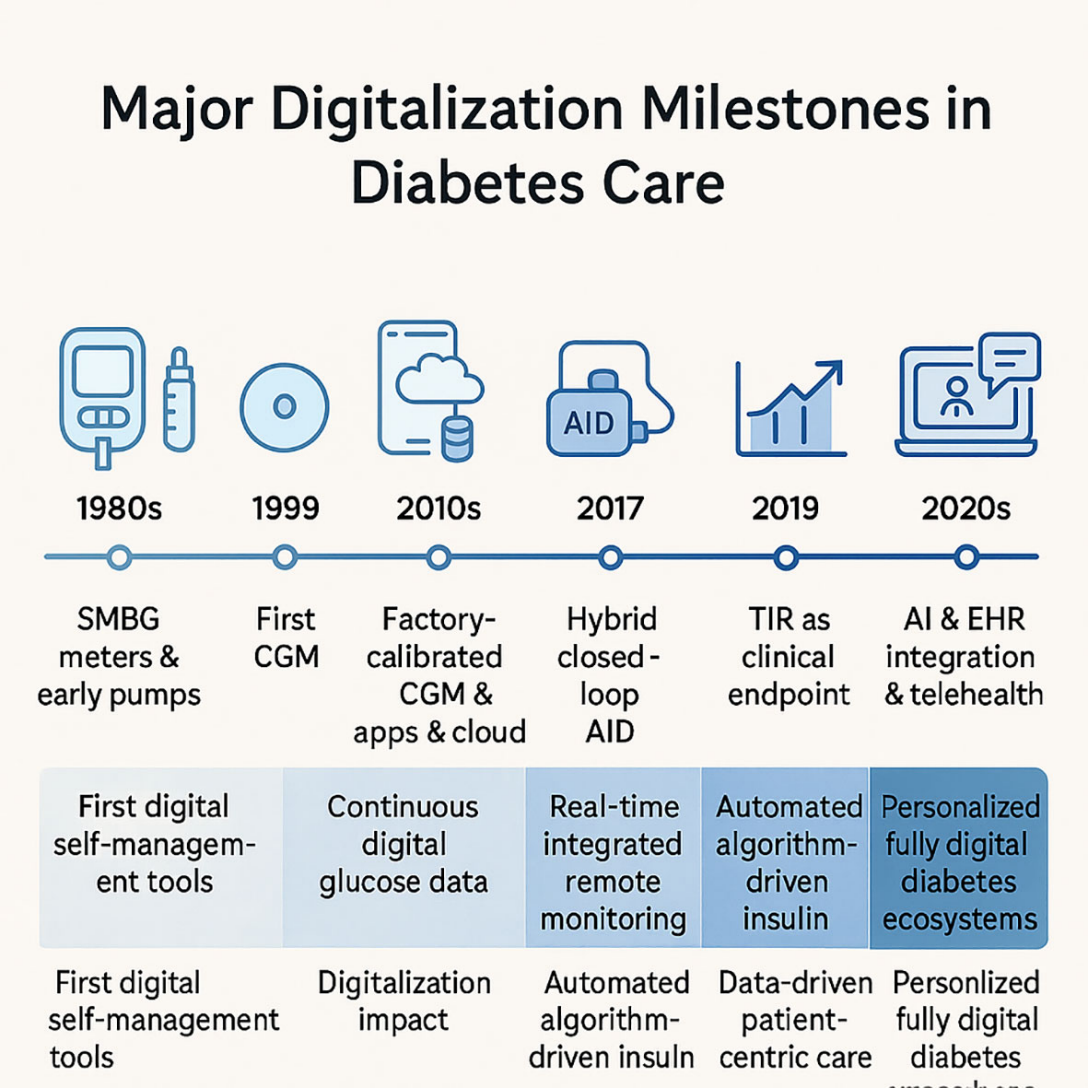
Key milestones in the digital transformation of diabetes care. This figure illustrates the major technological shifts that have driven the digitalization of diabetes management since the 1980s. The timeline progresses from the introduction of initial digital self-management tools, such as blood glucose meters, to the development of continuous glucose monitoring (CGM) and automated insulin delivery (AID) systems. It culminates in the current era, characterized by data-driven, patient-centric care using metrics like Time in Range (TIR) and the integration of Artificial Intelligence (AI) with telehealth and electronic health records (EHR) to create personalized, fully digital care ecosystems.

Over the past decade, diabetes care has shifted from standalone devices requiring significant manual input to integrated digital ecosystems (11). The rapid evolution of digital diabetes technology has progressed through several key phases, from early self-monitoring tools to today’s integrated, AI-driven ecosystems (**Figure 1**). Early tools, such as self-monitoring blood glucose (SMBG) meters and insulin pumps, have evolved into advanced, connected systems like factory-calibrated continuous glucose monitoring (CGM) systems, smart insulin pens, and automated insulin delivery (AID) devices (12, 13). These technologies enable real-time glucose monitoring, automated insulin dosing, and improved glycemic control, supported by strong evidence of their effectiveness and impact on quality of life (12, 14–17).

The COVID-19 pandemic further accelerated the adoption of telehealth and remote monitoring, enabling virtual consultations, asynchronous data review, and expanded access to care (18–21). During this period, CGM-derived metrics, such as time-in-range (TIR), gained prominence as clinical endpoints, complementing traditional HbA1c measures (22, 23).

Moving forward, the convergence of CGM, AID, smart pens, connected meters, telehealth, and decision-support systems is expected to further integrate diabetes management—provided that interoperability, data quality, and seamless electronic health record (EHR) integration are prioritized (12, 24–27). The frontier of diabetes care now lies in ensuring that these technological advances are equitably disseminated, scalable, and culturally competent, so that all individuals—regardless of background—can benefit from the digital revolution in diabetes treatment (28–30).

## 3 Challenges in adopting digital technologies

### 3.1 Patient barriers to adoption

Affordability remains a significant barrier to the widespread adoption of digital diabetes technologies. The costs of devices, data plans, and reliable internet access can limit access, particularly for underserved populations (31). Additionally, digital and health literacy challenges—such as difficulty with device setup, data interpretation, and troubleshooting—are common, especially among older adults (32). Physical and cognitive impairments further exacerbate these barriers (31), while concerns about data security and privacy may reduce trust and engagement with digital tools (33, 34). Beyond individual barriers, the rapid expansion of digital health technologies raises broader societal concerns. These include potential widening of health inequities for those unable to access these technologies, risks of algorithmic bias in AI-driven care recommendations, and environmental impacts from device manufacturing and energy-intensive data centers (35). Such considerations warrant attention as digital health solutions scale globally.

### 3.2 Physician barriers to integration

Healthcare providers also face challenges in integrating digital tools into clinical practice. Limited training, insufficient infrastructure, and the complexity of managing large volumes of data generated by digital devices are major obstacles (36, 37). These shortcomings can severely restrict the seamless implementation of digital solutions. Regulatory and privacy concerns, along with a lack of organizational support and clear implementation strategies, further hinder adoption (37, 38). Addressing these barriers requires targeted training programs, robust IT support, and incentives to encourage the integration of digital health technologies.

Together, these patient and provider challenges contribute to a persistent digital divide, wherein access to and effective use of digital diabetes technologies remain unevenly distributed across populations—most notably along generational, socioeconomic, and geographic lines. This divide not only limits the impact of technological innovation but also amplifies existing health disparities in diabetes care.

### 3.3 The digital divide: digital natives vs. digital immigrants

The digital divide remains a key obstacle to equitable diabetes care. This divide disproportionately affects underserved populations, such as those with lower socioeconomic status, rural residents, and older adults (39–41). Generational differences further exacerbate this gap. “Digital immigrants,” including older adults and many experienced healthcare professionals, often struggle with adopting and using digital tools, while “digital natives”—younger generations raised in a digital environment—possess inherent technological fluency (42, 43).

**Figure 2:**
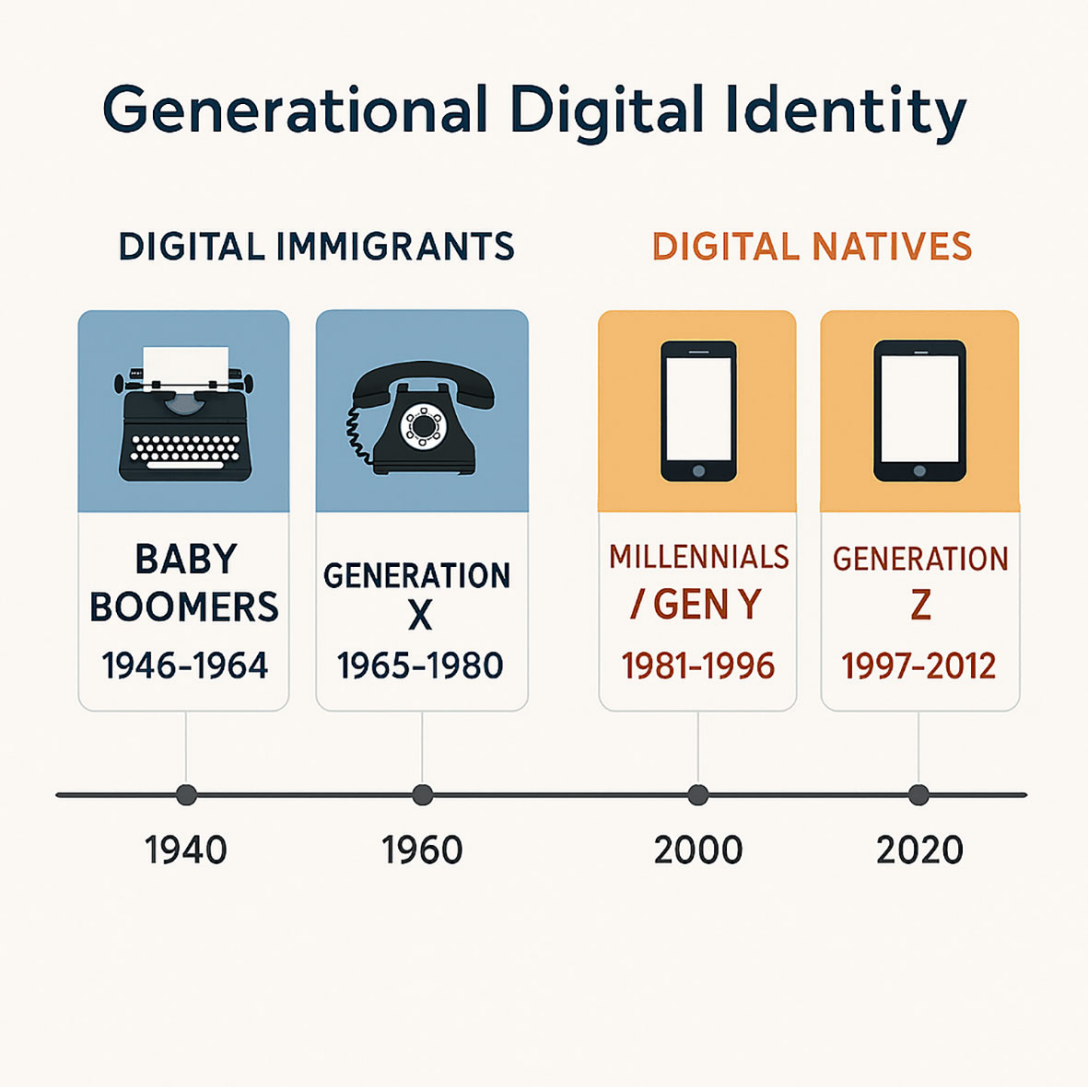
Generational digital identity.

Generational cohorts are often defined by the technological environment in which they grew up, shaping their comfort, familiarity, and fluency with digital tools (**Figure 2**) (44):

- Baby Boomers (1946–1964) (45): Digital immigrants who had to adapt to digital technologies later in life.
- Generation X (1965–1980) (46): Digital immigrants who witnessed the early adoption of personal computers and the internet.
- Millennials (Generation Y, 1981–1996) (72): Digital natives raised alongside the rise of the internet and mobile technologies.
- Generation Z (1997–2012) (47): Digital natives immersed in a fully digital world from birth.

These generational distinctions highlight the potential for intergenerational collaboration, with digital natives serving as mentors to help bridge the digital divide (**Table 1**).

**Table 1:** Parallel timeline- generational cohorts and diabetes technology milestones.

Year/Period	Diabetes technology milestone	Generational context (Digital natives vs\. Digital immigrants)
Pre-1980	- Insulin therapy (1921–1922) - Early SMBG meters (1970s–1980s)	Digital Immigrants predominate (Baby Boomers, Gen X)
1980–2000	- Electrochemical glucose meters (1987) - Early insulin pumps - First CGM (1999)	Digital Natives (Millennials) born (1981+) Immigrants adapt
2001	- “Digital native” term coined (Prensky, 2001)	Digital Natives (Millennials, Gen Z) enter childhood/adolescence
2004–2007	- Real-time CGM (2004) - Smart insulin pens (2007)	Natives: teens/young adults Immigrants: adults
2010s	- Factory-calibrated CGM - Mobile health apps - Early AID systems	Natives: young adults Immigrants: older adults
2017–2019	- Hybrid closed-loop (artificial pancreas) - TIR as clinical endpoint (2019)	Natives: workforce/health students Immigrants: main patient base
2020 (COVID)	- Telehealth & remote monitoring mainstreamed - CGM metrics in guidelines	Natives: digital mentors Immigrants: highest tech barriers
2020s	- Integration: CGM, AID, smart pens, telehealth, AI support	Natives: lead adoption/education Immigrants: need support

Quantitative evidence underscores the scope and persistence of these digital divides. For example, CGM adoption and sustained use are markedly lower among older adults, with some studies documenting a >50% reduction in uptake among those aged 65 and above compared to younger cohorts (40, 48). In a synthesis of digital intervention studies, 76% (16 of 21) reported significant within-group improvements in glycemic control (A1c reduction), while only about 41% (7 of 17) showed significant benefits between groups—highlighting persistent gaps in equitable outcomes (49). Additionally, digital divide issues—including device access, broadband connectivity, and digital literacy—were identified as barriers in up to five studies, disproportionately affecting rural, older, and low-literacy populations (39, 48). No included study found a population completely free of disparities, emphasizing the need for innovative, community-anchored solutions (50–52).

## 4 Strategies for advancing digital health equity

In response to persistent disparities in digital diabetes care, recent years have witnessed a growing emphasis on both equity and implementation science. Effective solutions require a multifaceted approach that integrates policy changes, culturally sensitive program design, and deep community involvement (41, 51, 52).

### 4.1 Policy reforms and systemic reforms

Policy initiatives, such as expanding coverage for digital health technologies and adopting value-based care models, can help reduce barriers to access. These systemic changes support the equitable adoption of digital tools across a wide range of patient populations (41, 51). Legislative measures such as the Affordable Care Act and Medicaid/Medicare expansion have played a crucial role in improving access to digital diabetes management resources (53, 54).

However, policy advances alone are insufficient. Achieving equity requires addressing other key determinants, including technology access, digital literacy, and robust support systems, to narrow the digital divide and ensure the benefits of digital health are distributed fairly.

### 4.2 Community engagement and participatory design

Engaging patients and local communities throughout the design, deployment, and evaluation of digital health solutions is critical for ensuring these interventions are both effective and equitable. By involving community members early, researchers and providers can better understand local needs, preferences, and barriers to adoption. Participatory design approaches play an important role in building trust, improving both cultural and linguistic relevance, and increasing the chances of successful adoption (41, 48). This is particularly important for vulnerable and underrepresented groups, who often face structural barriers that can limit their engagement with digital health tools (55).

### 4.3 Culturally competent interventions

Current guidelines emphasize the importance of designing digital health interventions that reflect the cultural values, communication preferences, and language needs of diverse communities (56). Adapting programs in this way helps build trust, encourages participation, and supports better adherence among people from different racial and ethnic backgrounds (55). For example, multilingual and culturally aligned digital platforms have been shown to enhance efficacy and sustain engagement in diabetes management across multiethnic populations (57).

Collectively, this body of evidence highlights that advancing digital diabetes care and addressing persistent disparities in access and outcomes cannot be achieved through a single intervention alone. Effective solutions require the integration of multiple determinants, including policy-driven coverage expansions, comprehensive digital health equity strategies, culturally tailored programs, and sustained community engagement.

## 5 Digital natives as change agents: a student-led solution

Empowering digital natives—especially university students in the health sciences—to serve as catalysts for digital transformation presents a promising and innovative strategy to bridge the digital divide in healthcare (58, 59). Digital natives can act as change agents for digital transformation by reframing the challenge from a lack of skills to an opportunity for intergenerational knowledge transfer. By leveraging their familiarity with technology and their emerging expertise in healthcare, these students can not only serve as learners but also as facilitators who help translate complex digital tools into accessible, practical solutions for diverse patient populations. This approach not only helps reduce the digital literacy gap but also fosters social responsibility, cultivates leadership skills, and builds a culture of support for digital health adoption among future healthcare professionals.

Students can play a vital role by providing hands-on training to older adults, equipping them to use diabetes technologies effectively. They can also collaborate with clinics and hospitals to deliver digital navigator services, assisting patients with technology setup and troubleshooting (60, 61). Implementation can include engaging student organizations in partnerships with local communities and establishing digital health literacy programs on university campuses that serve as hubs for improving access, skills, and ongoing support (62). In addition to patient support, students are positioned to assist healthcare professionals, many of whom feel unprepared to recommend or help patients with digital health tools due to limited training. Evidence indicates that healthcare providers face considerable obstacles in adopting new technologies, such as inadequate training, insufficient infrastructure, usability issues, and psychological resistance (36, 63).

Empowering students to teach patients and healthcare professionals through hands-on workshops, structured mentorship, and ongoing collaboration offers multifaceted benefits to all participants. Patients and professionals gain practical knowledge and skills to confidently navigate digital health technologies, while students themselves experience significant growth in their learning and expertise (64). When students take on the role of digital health educators, they reinforce their own understanding and practical abilities. This process not only strengthens their technical competency, but also provides valuable opportunities to develop communication, leadership, and problem-solving skills, which are essential in contemporary healthcare environments (65). The reciprocal nature of this educational model transforms students into organizational digital champions, encouraging intergenerational collaboration and creating a sustainable pathway for ongoing learning and teaching within the healthcare setting (44).

Participation in digital health education initiatives prepares students for future professional roles, ensuring they are equipped to contribute effectively to the rapidly evolving landscape of digital healthcare (66). A notable example of this approach is the Health Technology Navigators program, in which pre-health college students supported both patients and staff in safety-net clinics (67). This involvement led to measurable improvements, including increased patient portal enrollment and greater adoption of digital health tools. Furthermore, student-led clinics in allied health disciplines may demonstrate the value of experiential learning and interprofessional collaboration (68).

Recognizing the unique capabilities of this technologically fluent generation, student organizations can play a pivotal role. The establishment of student-led digital health literacy centers on university campuses could provide a structured platform for this work. These centers could develop and implement programs to:

- Train older patients in the community on how to use their specific diabetes technologies.
- Offer support sessions for healthcare professionals (digital immigrants) to build confidence with new clinical software and patient data platforms.
- Partner with clinics and hospitals to provide “digital navigator” services, assisting patients with technology setup and troubleshooting.

While preliminary reports and case studies suggest that student-led digital literacy initiatives may help bridge the digital divide in diabetes care, direct, high-quality evidence evaluating the effectiveness and long-term impact of these models remains limited. Acknowledging this gap, future research should focus on systematically assessing the outcomes of student-facilitated programs, including their influence on digital health adoption among both patients and healthcare professionals, as well as their broader effects on health equity and diabetes management.

## 6 Potential barriers and practical solutions for implementing student-led initiatives

While student-led digital health initiatives hold significant promise, several practical barriers must be addressed to ensure successful implementation.

Firstly, students themselves may require additional training to effectively teach digital health concepts and technologies to healthcare professionals. Regular, structured preparatory sessions and supervision by faculty or experienced mentors can help build this foundational competence (69, 70).

Secondly, resource allocation—including dedicated time, space, and access to relevant technologies—may be limited within healthcare and academic institutions. Securing institutional commitment and integrating these initiatives into existing professional development programs or curricula can help overcome these constraints (63, 71).

Thirdly, strong institutional support is critical to legitimizing student-led activities and ensuring collaboration with healthcare providers. Appointing organizational digital champions and establishing clear communication channels between students, academic leaders, and clinical staff can foster a supportive environment (36, 71). Furthermore, providing incentives or formal recognition for student participation can enhance engagement and sustainability (28, 71). By proactively addressing these barriers through targeted training, resource planning, and institutional endorsement, student-led initiatives can more effectively contribute to provider digital readiness and the equitable adoption of digital health technologies (63, 69, 70).

## 7 Conclusion

Bridging the digital divide in diabetes care requires a comprehensive approach that addresses the unique challenges faced by both patients and providers. Expanding access, enhancing digital literacy, investing in infrastructure, and fostering intergenerational collaboration are essential steps toward equitable adoption of digital health technologies. Ultimately, empowering the technologically fluent generation of “digital natives” to lead education and implementation offers a promising and sustainable strategy, transforming the generational divide into an opportunity for a more inclusive and effective digital health ecosystem.
